# Neurons generated shortly after birth encode the scent of early-life happiness

**DOI:** 10.1371/journal.pbio.3003890

**Published:** 2026-07-15

**Authors:** Chloé Guillaume, Elisa Galliano

**Affiliations:** 1 Department of Physiology, Development and Neuroscience, University of Cambridge, Cambridge, United Kingdom; 2 Homerton College, University of Cambridge, Cambridge, United Kingdom

## Abstract

Smells linked to joyful childhood events trigger vivid memories in adulthood, yet the mechanism behind their formation has remained elusive. This Primer explores a new study in PLOS Biology that shows that neurons generated around birth play a key role in encoding these olfactory memories.

## Teaser

Smells linked to joyful childhood events trigger vivid memories in adulthood, yet the mechanism behind their formation has remained elusive. A new *PLOS Biology* study shows that neurons generated around birth play a key role in encoding these olfactory memories.

*“[The sun] drew from the long frilled strips of seaweed pinned to the wall a smell of salt and weeds, which was in the towels too, gritty with sand from bathing.”* [1]—*Virginia Woolf, To the lighthouse*

If asked whether they have ever smelled dimethyl sulphide, geosmin, and bromophenol, most readers would likely draw a blank. Yet, when asked to sniff these compounds (the main components of the smell of the sea, wet sand, and iodine-rich algae), they would instantly recognize them, and the smells alone would likely transport them back to childhood summers spent playing on the shore more vividly than any words or photographs ever could [2]. Indeed, as has been expressed in novels and poems countless times, smells are especially powerful in shaping memory and emotional valence, not only through paired food associations (the famous Proust effect, where the smell and taste of a madeleine evoke memories of tea with a much-loved aunt), but also through statistical learning, the gradual accumulation of associations [3], as smells repeatedly co-occur with specific contexts and states. This phenomenon, arguably most powerful during childhood, is enabled both by repeated exposure to consistent odor–experience pairings (*e.g.*, recurring summer beach holidays linking the smell of the sea with positive experiences), and by the close anatomical connections between brain regions involved in olfaction, memory, and emotion. As a result, strong, long-lasting olfactory associations that can persist throughout life are formed. These are often tied not to explicit rewards (such as a toy or a sweet treat), but to a more diffuse sense of happiness and well-being.

Why does olfaction exert such a strong influence on how memories are formed, particularly early in life, and how are they later retained? This question can be approached through a three-sided prism of ethological function, brain wiring, and unique neurobiology. Ethologically, olfaction is a key early-life sense in both mice and humans, which are altricial species dependent on parental care. Mice are born blind and deaf and remain so into the second postnatal week, whereas olfaction is functional at birth and so supports essential early-life behaviors such as suckling and nest finding, making it the primary channel for neonatal experience. The perinatal period is also a critical window of olfactory plasticity [4], during which chronic exposure to odors can reshape odor representations in the brain, potentially reflecting not only sensory-driven plasticity but also statistical learning of the home environment. In terms of wiring, as hinted at above, olfaction has privileged access to memory, reward, and emotion circuits. The olfactory bulb, the brain’s first smell-processing station, sends outputs directly to the olfactory cortex and olfactory tubercle, and from there to the orbitofrontal cortex, amygdala, and hippocampus, largely bypassing the thalamic relay. This direct connectivity provides rapid access to systems central to emotional and mnemonic processing [5] and is likely especially engaged during early life when olfaction dominates experience. Neurobiologically, the olfactory bulb, like the hippocampus, retains lifelong neurogenic capacity, allowing continuous integration of new neurons that may support experience-dependent coding across life stages. This is particularly prominent in the perinatal period, when there is a marked increase in interneuron production from the subventricular zone. These newly generatedneurons, including granule cells among other interneuron subtypes, migrate and integrate into the olfactory bulb during early life [6].

**Fig 1:**
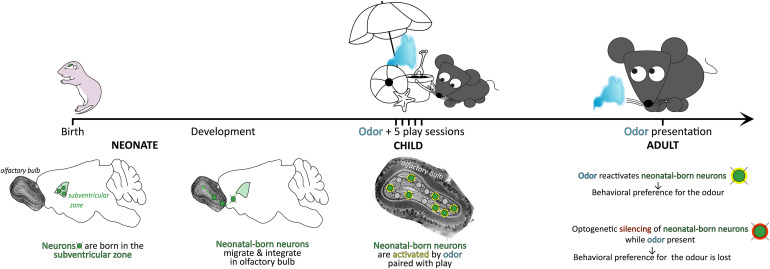
Neonatal-born granule cells encode positive odor-associated childhood memories. During the perinatal period, neurons are generated in the subventricular zone and migrate to the olfactory bulb, where they integrate into local circuits and become functionally mature. A subset differentiates into granule cells, the neuronal population investigated by Dejou and colleagues. During childhood, repeated exposure to an odor in a positive context, such a playpen, activates these perinatally generated granule cells. In adulthood, re-exposure to the same odor elicits a behavioral preference, suggesting the persistence of an olfactory memory formed during childhood. Silencing the neuronal population activated by the odor during childhood play abolishes this behavioral preference.

A new study published in the current issue of *PLOS Biology* by Dejou and colleagues [7] sheds light on how this childhood odor memory phenomenon is mechanistically implemented in the mammalian brain. The authors began with a human survey to establish the timing of childhood memory formation, recall, and long-term retention, then used this to guide the following mouse experimental work (Fig 1). In mice, they combined neuron birth-dating, behavioral tests, optogenetic manipulations, and cFos-based functional connectivity mapping to investigate how these memories are encoded and maintained. Mice who were exposed to an odor in a playful, positive, but not explicitly rewarding context during a childhood-equivalent period later showed a preference for that odor in early adulthood, which persisted into late adulthood if they continued to be re-exposed to the odor. In young adulthood, the memory was encoded in perinatally-generated granule cells in the olfactory bulb, and indeed, optogenetically silencing those specific neurons abolished mice’s preference for the odor. Moreover, early-adulthood mice showed strengthened connectivity between olfactory and reward-related brain regions. By contrast, late-adulthood recall was associated with increased connectivity with limbic rather than reward-related systems, indicating a shift in the underlying circuit mechanisms over time.

This study stands out for its originality in using a brief human study to inform the design of a mouse behavioral paradigm. The survey identified the timing and frequency of odor exposure that was important for memory formation in humans, and this timeline was generalized to developmentally equivalent periods in mice. This translational design enabled manipulation (arguably one of the study’s major strengths) via optogenetic targeting of the granule cell population, establishing a causal relationship between their activity and odor recall. Building on this strong foundation, further studies could disentangle perceptual and memory-related components and determine whether mice’s preference for the childhood-associated odor reflects episodic-like recall of early-life experience, a learned odor preference arising from repeated positiveexposure, or a combination of both. For instance, examining changes not only at the level of bulbar granule cells but also at earlier perceptual stages of the olfactory pathway, where olfactory sensory neurons and glomerular layer interneurons undergo lifelong regeneration and exhibit substantial plasticity in response to developmental odor enrichment [8], could help disentangle these mechanisms and bring the findings closer to those described in the human survey. Furthermore, the finding that perinatally-generated granule cells are engaged during childhood odor–experience pairings aligns well with previous work demonstrating that distinct bulbar interneurons cohorts can be recruited during pregnancy and removed after weaning, supporting state-dependent behavioral demands [9]. These perinatally-generated neurons may therefore be specialized for encoding early-life experiences with long-term behavioral relevance, although future work is needed to determine whether they differ in their physiological and morphological properties.

Another strength of the paper is that analysis of immediate early gene expression enabled characterization of functional connectivity across cortical, memory-related, reward, and olfactory-limbic regions. This extends the findings beyond the olfactory bulb and suggests widespread plastic reorganization of brain networks following early-life exposure and aging. To expand this framework further, it would be informative to also consider aversive/negative-valanced olfactory learning. It is well-established that olfactory cues can reliably elicit conditioned fear responses in adult mice [10], and recent work even suggests transgenerational inheritance of fear-related odors via biasing of stem cells in the olfactory epithelium [11]. It would be interesting to investigate whether similar mechanisms operate at the developmental stage examined by Dejou and colleagues, and whether negative memory traces rely on the same neural mechanisms and temporal dynamics, such as the need for repeated exposure during childhood as those described in their study, or whether they instead resemble more rapid, one-shot learning processes *(“By then Esthappen and Rahel had learned that the world had other ways of breaking men. They were already familiar with the smell. Sicksweet. Like old roses on a breeze”* [12]*, Arundhati Roy, The God of Small Things*). Addressing this question could help determine whether shared or distinct neural substrates underlie memories of positive childhood experiences and those of traumatic early-life experiences.
